# Antimelanoma Effects of *Alchemilla vulgaris*: A Comprehensive In Vitro and In Vivo Study

**DOI:** 10.3390/diseases12060125

**Published:** 2024-06-08

**Authors:** Sanja Jelača, Ivan Jovanovic, Dijana Bovan, Sladjana Pavlovic, Nevena Gajovic, Duško Dunđerović, Zora Dajić-Stevanović, Aleksandar Acović, Sanja Mijatović, Danijela Maksimović-Ivanić

**Affiliations:** 1 Department of Immunology, Institute for Biological Research “Siniša Stanković”—National Institute of the Republic of Serbia, University of Belgrade, Bulevar Despota Stefana 142, 11108 Belgrade, Serbia; sanja.jelaca@ibiss.bg.ac.rs (S.J.); dijana.draca@ibiss.bg.ac.rs (D.B.); 2 Center for Molecular Medicine and Stem Cell Research, Faculty of Medical Sciences, University of Kragujevac, Svetozara Markovića 69, 34000 Kragujevac, Serbia; ivanjovanovic77@gmail.com (I.J.); sladjadile@gmail.com (S.P.); gajovicnevena@yahoo.com (N.G.); 3 Institute of Pathology, School of Medicine, University of Belgrade, Dr Subotića 8, 11000 Belgrade, Serbia; dusko.dundjerovic@med.bg.ac.rs; 4 Faculty of Agriculture, University of Belgrade, Nemanjina 6, 11080 Belgrade, Serbia; dajic@agrif.bg.ac.rs; 5 Department of Dentistry, Faculty of Medical Sciences, University of Kragujevac, Svetozara Markovića 69, 34000 Kragujevac, Serbia; dr.acovic115@gmail.com

**Keywords:** lady’s mantle, ethanolic extract, *Alchemilla vulgaris* L., melanoma, tumor grade, metastasis, tumor microenvironment

## Abstract

Due to the rich ethnobotanical and growing evidence-based medicine records, the *Alchemillae herba*, i.e., the upper parts of the Lady’s mantle (*Alchemilla vulgaris* L.), was used for the assessment of antimelanoma activity. The ethanolic extract of *A. vulgaris* strongly suppressed the viability of B16F1, B16F10, 518A2, and Fem-X cell lines. In contrast to the in vitro study, where the B16F1 cells were more sensitive to the treatment than the more aggressive counterpart B16F10, the results obtained in vivo using the corresponding syngeneic murine model were quite the opposite. The higher sensitivity of B16F10 tumors in vivo may be attributed to a more complex response to the extract compared to one triggered in vitro. In addition, the strong immunosuppressive microenvironment in the B16F1 model is impaired by the treatment, as evidenced by enhanced antigen-presenting potential of dendritic cells, influx and activity of CD4^+^ T and CD8^+^ T lymphocytes, decreased presence of T regulatory lymphocytes, and attenuation of anti-inflammatory cytokine production. All these effects are supported by the absence of systemic toxicity. *A. vulgaris* extract treatment results in a sustained and enhanced ability to reduce melanoma growth, followed by the restoration of innate and adopted antitumor immunity without affecting the overall physiology of the host.

## 1. Introduction

Malignant melanoma is known as the most aggressive type of cancer resulting from the neoplastic transformation of melanocytes [1,2]. Although it accounts for only 1% of all skin malignancies, it is the leading cause of skin cancer-related deaths [3,4,5,6]. Following the approval of first- and second-generation immunotherapy agents which belong to the checkpoint blockade inhibitors, survival has improved significantly among these patients. Median survival of unresectable metastatic disease has been prolonged from 6–9 months to nearly 6 years for the combination of CTLA-4 and PD-1 blockade [7,8]. Despite these recent advances, many patients currently treated with immunotherapy experience disease progression due to initial or acquired resistance. The development of drugs and new therapeutic strategies that address these issues, therefore represents a major challenge, opening the space for complementary or alternative medicine. On the list of non-conventional approaches in medicine, natural substances derived from plants occupy the leading position [9]. Having in mind that a substantial number of official chemotherapeutics are drugs isolated from plants or synthesized on the basis of plant biomolecules, medicinal herbs can be considered a powerful source of potential drugs that has been frequently investigated in recent decades [10,11]. Besides the fact that pure compounds represent an accurate and reproducible system for evaluating biological effects, the general impression arising from scientific and ethnobotanical experiences is that total extracts, more specifically their lipophilic fractions, have a greater potential for tumor growth suppression from individual components due to interactions of polyphenolic molecules [12]. This observation can be compared to official protocols where several drugs with different intracellular signaling inputs are combined to avoid the emergence of resistance [13,14]. Relating rich ethnobotanical records and high phenolic content reported for the Balkan populations of the *Alchemilla vulgaris* L. *sensu latiore*, commonly known as Lady’s mantle, the Alchemillae herba was selected for a study performed in line with the evidence-based medicine [15,16,17]. We have previously shown that extract from the aerial part of the plant, obtained by different extraction procedures, has a high potential to decrease the viability of numerous tumor cells in vitro, such as tumors of the female reproductive organs (HeLa and A2780), human prostate (PC-3), breast (MCF-7), lung (A549), melanoma (A375) and colon (HCT116) cell lines [16,17]. Moreover, even the root extract showed remarkable cytotoxic activity against human prostate cancer (PC-3), breast cancer (MCF-7), and human colorectal cancer (Caco2) cell lines [18]. Since the effect of an isolated compound is not equivalent to the orchestrated influence of the extract mixture, the results of certain treatments observed in cell culture are often far from those determined in vivo. This is particularly pronounced in the syngeneic model where all aspects of the immune response are introduced. Moreover, the influence of microenvironmental factors is not the same in different stages of the disease, so the effects of treatment can differ significantly depending on the degree of disease progression. 

Taking all into consideration, the present study evaluates for the first time the potential of *A. vulgaris* extract to suppress the viability of two different generations of B16 cells- F1 and F10 in vitro and in vivo, offering the opportunity to compare its efficiency depending on the differentiation status and the microenvironment [19,20].

## 2. Materials and Methods

### 2.1. Reagents and Cells

RPMI-1640 culture medium, Dulbecco Modified Eagle Medium (DMEM) high glucose culture medium, and fetal bovine serum (FBS) were acquired from Capricorn Scientific GmbH (Ebsdorfergrund, Hessen, Germany). Dimethyl sulfoxide (DMSO), propidium iodide (PI), Triton X-100, RNase, phosphate-buffered saline (PBS), 3-methyl adenine (3-MA), carboxyfluoresceindiacetate succinimidyl ester (CFSE), acridine orange (AO), collagenase I, ethylenediaminetetraacetic acid (EDTA), DNase I, trypsin, trichloroacetic acid (TCA), TRIS-HCL, Glutathione (GSH), N-Acetyl-L-cysteine (NAC), and sulforhodamine B (SRB) were bought from Sigma-Aldrich (St. Louis, MO, USA). Dihydrorhodamine 123 (DHR) was from Thermo Fisher Scientific (Waltham, MA, USA). The Penicillin Streptomycin solution was purchased from Biological Industries (Cromwell, CT, USA). 3-(4,5-dimethythiazol-2-yl)-2,5-diphenyltetrazolium bromide (MTT), and bovine serum albumin (BSA) were obtained from AppliChem (Maryland Heights, MO, USA). Fluorescein-di-β-D-galactopyranoside (FDG) was from Abcam (Cambridge, UK). Paraformaldehyde (PFA) was acquired from Serva (Heidelberg, Germany). Annexin V-FITC (AnnV) was from BD Pharmingen (San Diego, CA, USA). Apostat was acquired from R&D Systems (Minneapolis, MN, USA). LysoTracker™ Red DND-99 was obtained from Invitrogen (Waltham, MA, USA). Murine melanoma (B16F1, and B16F10) and murine embryonic fibroblasts (NIH/3T3) cell lines were obtained from American Type Culture Collection (Rockville, MD, USA). Human immortalized keratinocyte (HaCaT) cell line was obtained from CLS Cell Lines Service (Eppelheim, Germany). The human melanoma (518A2) cell line was a kind gift from Prof. Dr. Habil. and Dr. h.c. Goran Kaluđerović, Hochschule Merseburg, University of Applied Sciences, Merseburg, Germany. The human melanoma (Fem-X) cell line was a kind gift from Dr. Milena Čavić, Institute for Oncology and Radiology of Serbia, Belgrade, Serbia.

Cell lines (B16F1, B16F10, 518A2, and Fem-X) were cultivated in HEPES-buffered RPMI-1640 medium, while NIH/3T3, and HaCaT cell lines were cultivated in DMEM medium, both mediums previously supplemented with 10% heat-inactivated FBS, 2 mM L-glutamine, 0.01% sodium pyruvate, and antibiotics (penicillin (100 units/mL) and streptomycin (100 μg/mL)) (medium for cultivation). Cells were kept under standard growing conditions at 37 °C in a humidified atmosphere with 5% CO_2_. The density of B16F1 and B16F10 cells in 96-well plates was 3 × 10^3^ cells/well and 2 × 10^3^ cells/well, while for flow cytometric analyses in 6-well plates 1.5 × 10^5^ and 1 × 10^5^ cells/well respectively. The density of 518A2, Fem-X, NIH/3T3, and HaCaT cells in 96-well plates was 5 × 10^3^ cells/well.

The chemical content of the ethanolic extract derived from *Alchemilla vulgaris* L., employed in this research, as well as the extraction methodology, were previously documented [17]. Harvested from its native habitat in the moderately humid hilly mountainous grasslands of southeastern Serbia, the plant material, gathered during the flowering phase, was meticulously identified and archived as voucher specimens (No. RS-120718-1). The binary ethanol–water solvent (70% ethanol) was used as a green and universal solvent providing the highest yield of Lady’s mantle polyphenols, mainly phenolic acids, flavonoids, flavanones, and tannins [16]. Moreover, in the Balkans, the Achemilla tincture (ethanolic extract) is preferred over the consumption of its mono-component herbal tea (decoct). Following two-hour extraction, the chemical analysis of the resultant solid, i.e., vacuum-evaporated extract was conducted by UHPLC-HRMS, revealing the presence of 45 compounds, predominantly flavonol and flavone glycosides, notably derivatives of quercetin and kaempferol [17]. 

The obtained *A. vulgaris* ethanolic extract without solvent was used for the preparation of stock solution in pure DMSO (100%) to reach the concentration of 200 mg/mL. Working solutions were made from the stock solution by further dissolving in the medium for cultivation. The highest concentration of DMSO in working solutions did not exceed 0.1% for in vitro and 4% for the in vivo experiments.

### 2.2. Animals

Six-to-eight-week-old C57BL/6 female mice were obtained from the animal facility at the Institute for Biological Research “Siniša Stanković”—National Institute of the Republic of Serbia, University of Belgrade (Belgrade, Serbia). All animals were kept under standard laboratory conditions (nonspecific, pathogen-free), with unlimited access to food and water. The study protocol and handling of animals were in agreement with the rules of the European Union and approved by the local Institutional Animal Care and Use Committee (IACUC) and the European Community guidelines (EU Directive 2010/63/EU for animal experiments). Experimental protocols were approved and granted by the national licensing committee at the Department of Animal Welfare, Veterinary Directorate, Ministry of Agriculture, Forestry and Water Management of the Republic of Serbia (permission No. 323-07-12008/2020-05).

### 2.3. Colorimetric Assays for Cellular viability (MTT and SRB)

In order to evaluate the effect of *A. vulgaris* L. ethanolic extract on the viability of tumor cells, SRB and MTT assays were used. B16F1, B16F10, 518A2, Fem-X, NIH/3T3, and HaCaT cells were seeded overnight and exposed to a wide range of concentrations (3.13–200 µg/mL) of *A. vulgaris* extract for 72 h.

Afterward, for the SRB assay, cells were fixed using 10% of TCA for 2 h at 4 °C. After washing with distilled water, cells were stained for 30 min at room temperature (RT) with 0.4% SRB solution. Finally, cells were washed with 1% acetic acid and dried overnight. The dye was dissolved in 10 mM TRIS buffer. After 20 min of incubation at RT, the absorbance was measured at 540 nm with the reference wavelength at 670 nm. Results were expressed as a percentage of untreated cells (control) and all experiments were repeated in triplicate.

Alternately, at the end of the treatment, cells were incubated with MTT solution (0.5 mg/mL) for 90 min and the formed formazan crystals were dissolved by the addition of DMSO in cell culture. The absorbance was measured with an automated microplate reader at 540/670 nm while the results were expressed as a percentage of untreated cells (control). All experiments were repeated three times.

To estimate whether the production of ROS/RNS underlies the potential tumoricidal action of *A. vulgaris* extract, concomitant treatment of *A. vulgaris* extract and antioxidants GSH or NAC was performed. B16F1 and B16F10 cells were exposed to an IC_50_ dose of *A. vulgaris* extract in parallel with 0.8 mM GSH, or 1.25 mM NAC, and cell viability was evaluated after 72 h using MTT and SRB assays.

### 2.4. Detection of Apoptosis, Activation of Caspases, and Autophagy

B16F1 and B16F10 cells were seeded overnight and exposed to an IC_50_ dose (40 µg/mL and 80 µg/mL, respectively) of *A. vulgaris* extract for 72 h. 

For detection of apoptotic cell death, melanoma cells (B16F1, and B16F10) were stained with (15 μg/mL) Annexin V-FITC and (15 μg/mL) PI for 15 min at RT, protected from the light. Finally, cells were resuspended in an AnnV-binding buffer (ABB) and analyzed by flow cytometry using CyFlow^®^ Space (Partec, Munster, Germany). Apostat staining was used for the detection of total caspase activity and after the treatment cells were washed, trypsinized, and incubated with 0.5 µg/mL pan-caspase inhibitor Apostat for 30 min at 37 °C according to the manufacturer’s instructions. Afterward, cells were washed with PBS, resuspended, and analyzed as described above. To evaluate the presence of autophagy, two staining protocols were applied, supravital acridin orange (AO) and LysoTracker Red (Waltham, MA, USA) [21]. Depending on the pH, AO accumulates in acidic organelles (autolysosomes), where it is protonated and trapped, forming aggregates that fluorescence bright red. Similarly, LysoTracker selectively marked autophagosomes and acidic endo/lysosomal compartments. Cells were stained with AO dye (10 μM) for 15 min at 37 °C, washed, and resuspended in PBS before analysis. Alternatively, B16F1 cells were stained with LysoTracker Red (50 nM) for 30 min at 37 °C. After the incubation period, cells were washed with PBS, resuspended, and analyzed by flow cytometry. In order to specify the role of detected autophagy (cytoprotective vs. cytodestructive), combined treatment of *A. vulgaris* extract and autophagy inhibitor 3-MA was performed. B16F1 cells were exposed to an IC_50_ dose of *A. vulgaris* extract in the presence/absence of 1 mM concentration of 3-MA for 72 h and cell viability was evaluated with SRB assay.

### 2.5. Measurement of Reactive Oxygen and Nitrogen Species (ROS/RNS) Generation

Before seeding, melanoma cells (B16F1, and B16F10) were stained with redox-sensitive dye DHR 123 for 20 min (1 µM) at 37 °C and treated with an IC_50_ dose (40 µg/mL for B16F1 and 80 µg/mL for B16F10) of *A. vulgaris* extract. After 72 h, cells were washed with PBS, trypsinized, and analyzed by flow cytometry (CyFlow^®^ Space (Partec, Munster, Germany)).

To estimate whether the production of ROS/RNS underlies the potential antitumor action of *A. vulgaris* extract, melanoma cells (B16F1, and B16F10) stained with DHR 123 were exposed to IC_50_ dose (40 µg/mL for B16F1 and 80 µg/mL for B16F10) of *A. vulgaris* extract alone or in combination with GSH (0.8 mM) or NAC (1.25 mM). After 72 h cells were washed with PBS, trypsinized, and analyzed as mentioned above.

### 2.6. Detection of Cell Proliferation

For detection of cell proliferation, melanoma cells (B16F1, and B16F10) were prestained with 1 μM CFSE solution for 10 min at 37 °C. After incubation, cells were washed, seeded, and then exposed to an IC_50_ dose of *A. vulgaris* extract (40 µg/mL for B16F1 and 80 µg/mL for B16F10). After 72 h, cells were washed, trypsinized, dissolved in PBS, and analyzed using flow cytometry (CyFlow^®^ Space (Partec, Munster, Germany)).

### 2.7. Senescence Determination

B16F1 cells were cultivated in 6-well plates and treated with an IC_50_ dose (40 µg/mL) of *A. vulgaris* extract for 72 h. Afterward, cells were washed with PBS, trypsinized, and incubated with 1 mM FDG at 37 °C for 1–2 min. The reaction was stopped by adding 900 µL of ice-cold RPMI medium. Cells were analyzed within 1 h after staining by flow cytometry (CyFlow^®^ Space (Partec, Munster, Germany)).

### 2.8. Cell Cycle Analysis

In order to evaluate the distribution of B16F1 and B16F10 cells within cell cycle phases, cells were cultivated in 6-well plates overnight and treated with IC_50_ concentration of *A. vulgaris* extract for 72 h. Afterward, cells were washed with PBS, trypsinized, and fixed in ice-cold 70% ethanol overnight at 4 °C. Finally, cells were washed in ice-cold PBS and incubated with 10 µg/mL PI and 0.1 mg/mL RNase for 45 min at 37 °C. After incubation period cells were analyzed using flow cytometry (CytoFLEX Flow Cytometer, Beckman Coulter, Life Sciences, Indianapolis, IN, USA).

### 2.9. PI Staining on Chamber Slides

For evaluation of the morphological signs of apoptosis B16F1 and B16F10 cells were seeded overnight in chamber slides. Afterward, cells were treated with an IC_50_ concentration of *A. vulgaris* extract for 72 h. At the end of incubation, cells were then fixed with 4% PFA for 15 min at RT, and subsequently stained with a solution of PI 50 μg/mL, 0.1 mM EDTA pH 8.0, 0.1% Triton X-100, and 85 μg/mL RNase in PBS for 1.5 min. Between all steps, cells were washed several times in PBS. At the end, a fluorescent mounting medium was added, and the analysis was conducted using Zeiss AxioObserver Z1 inverted fluorescence microscope (Carl Zeiss AG, Oberkochen, Germany) at a magnification of 400×.

### 2.10. Induction of Solid Melanoma and Experimental Treatment

Tumors were induced by subcutaneous (sc.) implantation of 1.8 × 10^5^ B16F1 or 2 × 10^5^ B16F10 cells in the dorsal right lumbosacral region of syngeneic C57BL/6 female mice. Treatment with 50 mg/kg *A. vulgaris* extract in 4% DMSO/PBS (i.p.) started when tumors became palpable. The treatment was applied for 5 consecutive days with a break of 2 days in between. The control group received the solvent in the same regimen. Mice were sacrificed when tumor volume reached the maximally allowed value in control animals. Tumor size was measured using a caliper and volume was calculated with the formula: (length × width^2^) × 0.52.

### 2.11. Histopathology

Tumors, kidneys, and livers of sacrificed animals were macroscopically examined and fixated in 10% buffered formalin of neutral pH value, for 24 h. Tissues were sectioned through the largest tissue plane and additionally fixated for 24 h. Processing of tissues was done in an automatic tissue processor (Milestone SRL LOGOS ONE, Sorisole, BG—Italy). Tissue was further embedded in paraffin blocks on the embedding console (SAKURA Tissue-Tek TEC 5, Sakura Finetek, CA, USA). Tissue slices (4 µm) were stained by hematoxylin and eosin and analyzed by Olympus BX43 microscope (OLYMPUS EUROPA HOLDING GMBH, Hamburg, Germany). The digitalization of all slides was additionally carried out with a Leica Aperio AT2 slide scanner (Leica Biosystems, GmbH, Nussloch, Germany) for analysis and documentation purposes. Virtual slides generated from Leica Aperio AT2 were morphometrically analyzed with a Leica AperioImageScope (version 12.4.6, Leica Biosystems, GmbH, Nussloch, Germany) and with FIJI-ImageJ 1.51j8 software.

### 2.12. Immunofluorescence PCNA Detection

B16F1 tumor tissue slides were deparaffinized and rehydrated before epitope retrieval by microwaving in citrate buffer. Blockade of nonspecific binding was carried out with 5% BSA at RT, for 30 min, and subsequently, tissues were incubated overnight, at 4 °C with rabbit polyclonal anti-PCNA antibody (eBioscience, San Diego, CA, USA, 1/100). As a secondary antibody, anti-rabbit Alexa Fluor 488 (Invitrogen, Thermo Fisher Scientific, Waltham, MA, USA, 1/400) was applied for 2 h at RT. After washing, PI counterstaining was performed (1 mg/mL, for 5 min). At the end, all samples were mounted with Fluoromounth G (Southern Biotech, Birmingham, AL, USA) and examined with an SP5 confocal microscope (Leica Microsystems CMS GmbH, Wetzlar, Germany).

### 2.13. Preparation of Single-Cell Suspensions

Single-cell suspensions of primary tumors and spleens were obtained as previously described [22]. After isolation, primary B16F1 tumors were shredded and then placed in 5 mL of RPMI medium containing 1 mg/mL collagenase I, 1 mM EDTA, and 2% FBS to perform enzymatic digestion. After incubation for 2 h at 37 °C, 10 mL of 0.25% trypsin was added and incubated for 3 min. Further, the cells were treated with DNase I solution for 1 min and filtered through a 40 μm nylon cell strainer BD Biosciences (San Jose, California, SAD). Single-cell suspensions from spleens were obtained by mechanical disruption.

### 2.14. Flow Cytometric Analysis

Single-cell suspensions were labeled with fluorochrome-conjugated monoclonal antibodies: anti-mouse CD3, CD4, CD8, CD25, CD86, CD40, CD49b, PD-1, CD11c, and MHC II antibodies (BD Pharmingen; BioLegend, San Diego, CA, USA; eBiosciences, San Diego, CA, USA) or with isotype-matched control and analyzed on a FACSCalibur (BD Biosciences, San Jose, CA, USA, SAD) using CELLQUEST 5.1 software (BD Biosciences, San Jose, CA, USA, SAD). 

For intracellular staining, cells were stimulated with Phorbol 12-myristate13-acetate (50 ng/mL)/ionomycin (500 ng/mL) (Sigma-Aldrich, St. Louis, MO, USA) and GolgyStop (BD Pharmingen, San Diego, CA, USA) for 4 h at 37 °C, 5% CO2, stained with fluorochrome-labeled anti-mouse antibodies specific for CD4 or CD8, fixed and permeabilized with a Cytofix/Cytoperm solution. Intracellular staining was performed using monoclonal antibodies: CD107a, perforin, IFN-γ, IL-10, TNF-α and Foxp3 (BD Pharmingen; BioLegend, San Diego, CA, USA; eBiosciences, San Diego, CA, USA) or appropriate negative controls. Cells were analyzed with the FACSCalibur Flow Cytometer (BD Biosciences, San Jose, CA, USA), and analysis was conducted with FlowJo^TM^ 10.7.2. software.

### 2.15. Statistical Analysis

For evaluation of statistical significance between groups and for further data analyses, the package Statistica 12 (Informer Technologies, Inc., Los Angeles, CA, USA) was used and *p*-values of less than 0.05 were considered significant. The non-parametric Mann–Whitney test was used for the analysis of in vivo results.

## 3. Results

### 3.1. A. vulgaris Ethanolic Extract Downregulates Melanoma Cell Viability and Suppresses Tumor Cell Growth in Both, Low- and High-Grade Syngeneic Models of Primary Tumor

**Figure 1:**
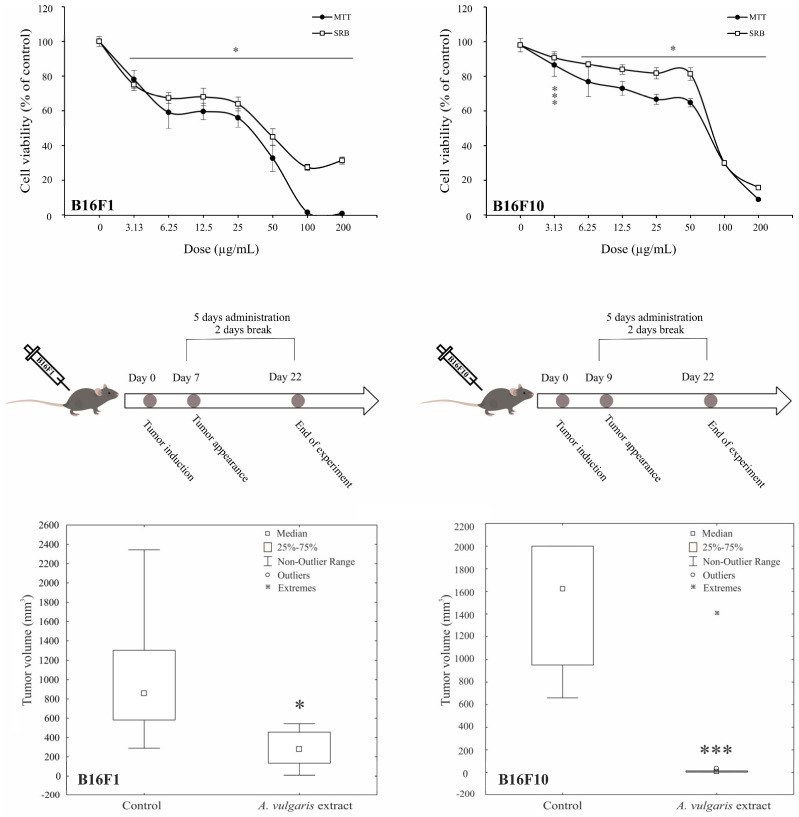
*A. vulgaris* extract decreases melanoma cell viability in vitro and suppresses tumor cell growth in vivo. B16F1 and B16F10 cells were treated with a wide range of concentrations of *A. vulgaris* extract and viability assays (MTT and SRB) were performed after 72 h. All data are presented as mean ± SD from one representative out of three independent experiments and statistically significant were considered *p* values less than 0.05, compared to controls (upper panel). Primary tumors were induced by sc. inoculation of B16F1 or B16F10 cells into C57BL/6 mice and treated with 50 mg/kg of *A. vulgaris* extract (n = 10 animals per group). For evaluation of statistical significance between groups in in vivo experiments, the non-parametric Mann–Whitney test was used (lower panel). * *p* < 0.05; *** *p* < 0.001.

Antimelanoma potential of *A. vulgaris* ethanolic extract was tested in two B16 melanoma cell lines of different invasive potential (F1 and F10), and two human melanoma cell lines (518A2, and Fem-X), and the cell viability was determined upon 72 h using MTT and SRB assays. As presented in Figure 1 and Figure S11, *A. vulgaris* extract decreased the number of viable cells in all cell cultures in a dose-dependent manner with a slightly more profound effect obtained by MTT than SRB measurements. Visual assessment of the cultures exposed to the extract revealed that values gained by SRB more precisely reflected the number of viable cells, indicating that cell respiration prior to viability is affected by the treatment, and thus IC_50_ values calculated from SRB were selected as more convenient for further investigation (Table 1). As expected according to their more aggressive phenotype, B16F10, showed lower sensitivity to applied treatment in comparison to its F1 counterpart. Additionally, the cell viability of primary peritoneal exudate cells [17], as well as murine embryonic fibroblasts (NIH/3T3), and human immortalized keratinocytes (HaCaT) was preserved after treatment with *A. vulgaris* extract (Figure S2) indicating selectivity of the treatment toward malignant phenotype.

Having in mind all the limitations of in vitro setting in the evaluation of separated compounds/mixtures or extracts’ potential to limit cancer cell growth, *A. vulgaris* efficacy was further explored using both- syngeneic models of low and highly aggressive primary solid melanomas induced by sc. inoculation of B16F1 and B16F10 cells, respectively. This approach enables examination of the tumor-suppressive potential of extracts from a platform that, unlike in vitro studies, but also the xenograft animal model, includes the fully preserved tumor microenvironment with all aspects involved in the tumor/host interaction, which makes it the most similar to human disease. Animals were treated daily with 50 mg/kg of *A. vulgaris* extract and after 22 days tumor volume was estimated. As shown in Figure 1 and Figure S11, the *A. vulgaris* extract significantly decreased tumor volume in both tumor models, apart from the initial differences in cell phenotype and, accordingly primary tumor consistency and invasiveness. Surprisingly and oppositely to data obtained in vitro, the effect was even more noticeable in the highly invasive B16F10 model (B16F1 *p* = 0.004; B16F10 *p* = 0.000491) underlining the common role of the microenvironmental factors in response to therapy and pointing out the limited insights that comes from in vitro research.

### 3.2. A. vulgaris Extract Manifests Dissimilar Mode of Action in B16 Melanoma Cell Lines of Different Grades In Vitro

**Figure 2:**
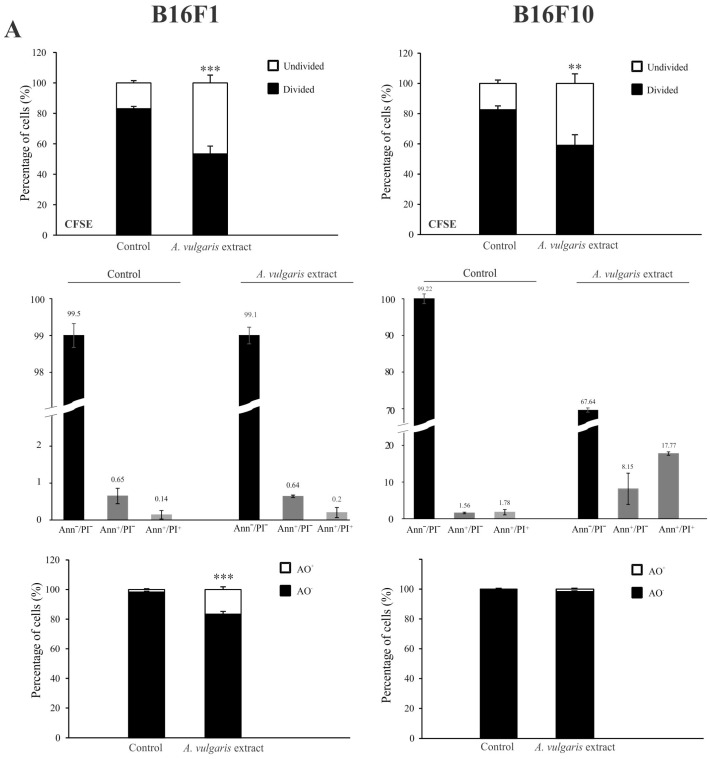

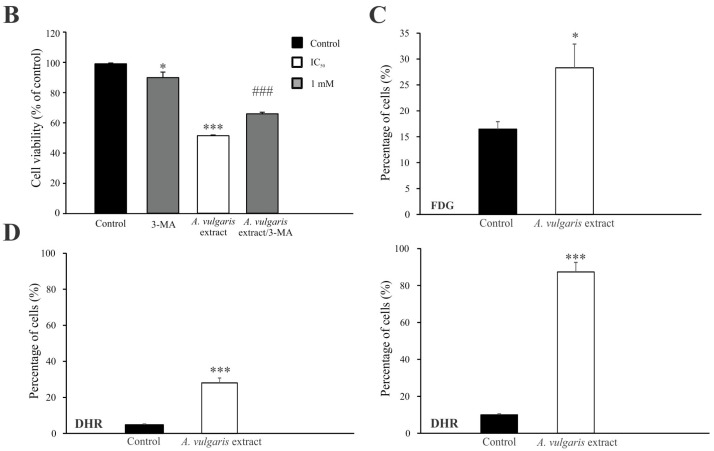
*A. vulgaris* extract exhibits different modes of action on B16F1 and B16F10 cell lines. Cells were treated with an IC_50_ dose of *A. vulgaris* extract for 72 h. Cellular proliferation (CFSE), apoptosis (Ann/PI), and autophagy (AO) were detected by corresponding staining followed by flow cytometry analysis (**A**). Data are presented as mean ± SD from three independent experiments. B16F1 cell viability after combined treatment with *A. vulgaris* extract and autophagy inhibitor 3-MA (1 mM) was assessed by SRB assay (**B**), * *p* < 0.05; ** *p* < 0.01; *** *p* < 0.001 compared to control and ### *p* < 0.001 comparing to *A. vulgaris* extract treatment. Cellular senescence was analyzed using FDG staining (**C**), while production of ROS/RNS species was detected by redox sensitive dye (DHR123) (**D**) and subsequent flowcytometric evaluation.

Flow cytometric analysis of proliferation rate and presence/characteristics of different types of cell death in B16F1 and B16F10 cultures exposed to IC_50_ concentrations of *A. vulgaris* in vitro revealed significant differences between F1 and F10 generation in response to the treatment, that can be ascribed to the phenotype changes obtained during the process of tumor progression. To estimate the cell division in response to the treatment, the B16F1 and B16F10 cells were stained with specific dye CFSE, and subsequently exposed to an IC_50_ dose of *A. vulgaris* extract for 72 h. The obtained results showed that *A. vulgaris* extract increased the percentage of undivided cells in both cell lines, with a more profound effect on the less invasive form (Figure S3 and Figure 2A, upper panel). In parallel, significant arrest in the S phase of the cell cycle was detected in both tested cell lines, while the tendency of hypodiploid cell accumulation was visible only in B16F10 cultures exposed to the treatment (Figure S4). Accordingly, Ann/PI double staining revealed that *A. vulgaris* extract led to apoptotic cell death of the highly invasive F10 cells, with mostly late apoptotic cell accumulation after 72 h, recognized by the Ann^+^/PI^+^ profile (Figure S3 and Figure 2A, middle panel). Illustration of the mentioned effect was obvious on fluorescence microscopy where irregular nuclei shape, shrunk nuclei, apoptotic bodies, and sporadic micronuclei were detected (Figure S5). Nevertheless, apart from intensive apoptosis, caspase activation was not observed (Figure S6). Oppositely to the higher sensitivity of B16F1 vs. B16F10 in vitro, the lack of early and late apoptotic cells in B16F1 cultures upon 72 h long treatment with *A. vulgaris* extract was noted (Figure S3 and Figure 2A, middle panel). The microscopical analysis of nuclear morphology discovered the presence of large and elongated nuclei that together with decreased B16F1 cell density were in line with previous statements about the absence of apoptosis (Figure S5). On the other hand, intensive autophagy, measured by AO and LysoTracker Red staining was noted only in the cultures of B16F1 cells (Figures S3 and S7, and Figure 2A, lower panel) but not B16F10. Since autophagy possesses a dual role and can oppose or mediate the cytotoxicity of the treatment, to specify its contribution to drug effectiveness, 3-MA, a specific inhibitor of autophagy, was applied. Namely, 3-MA blocks autophagosome formation and thus leads to autophagic process failure [23]. The inhibition of the autophagic process using this inhibitor confirmed the involvement of the autophagic process in the viability decrease triggered by the tested plant extract (Figure 2B). In addition, moderate cell senescence determined by FDG staining in vital cell fraction (Figure 2C), might further explain the good effect of the extract in B16F1 cell culture. All observed effects were in correlation with enhanced production of ROS/RNS determined by DHR staining, with a more noticeable effect in invasive clone (Figure 2D). In order to evaluate whether intensified production of ROS/RNS was responsible for the antitumor action of *A. vulgaris* extract, they were eliminated by antioxidants, GSH, or NAC. N-acetylcysteine provides a supply of cysteine necessary for GSH synthesis and replenishment [24]. The obtained results showed that concomitant treatment with *A. vulgaris* extract with GSH or NAC, significantly recovered the viability of B16F1 and B16F10 cells, in comparison to cells exposed to IC_50_ dose of *A. vulgaris* alone (Figure S8). In addition, flow cytometric analysis revealed that the amount of ROS/RNS triggered by the tested extract was remarkably reduced in the presence of GSH or NAC, confirming their importance as mediators of the tumoricidal action of the extract (Figure S9).

### 3.3. A. vulgaris Extract Promoted Tumor Shrinkage in Both Low-End High Aggressive Syngeneic Models through Unexpectedly Different Routes in Comparison to Those Determined In Vitro

**Figure 3:**
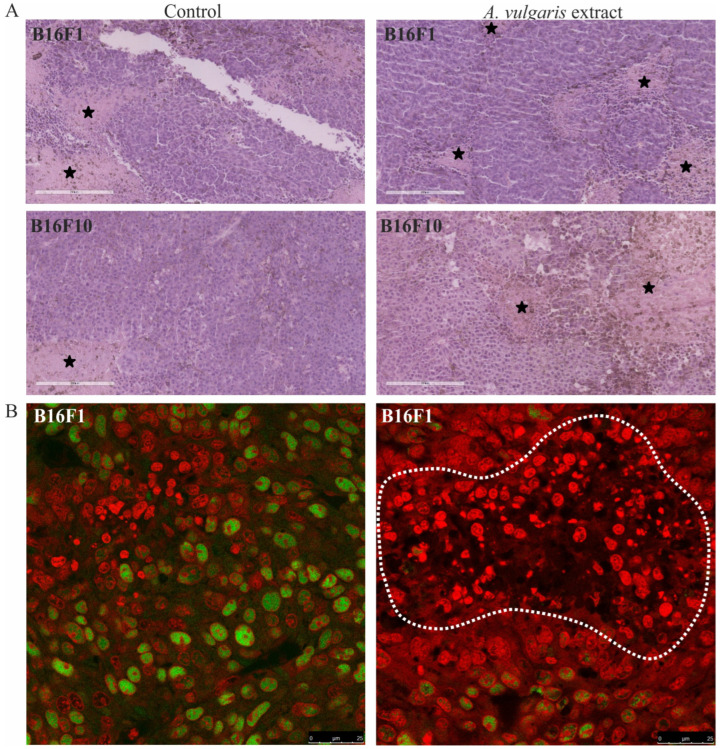
Representative micrographs of the most significant histopathological alterations noted in the B16F1 and B16F10 models. (**A**) H/E staining, scale bar 200 µm. Black stars are areas of tumor necrosis. (**B**) PCNA immunoexpression (green) in melanoma tissue of control animals (left) and animals treated with *A. vulgaris* extract (right). Scale bar 25 µm. The apoptotic area is surrounded by a white interrupted line.

The histopathological analysis of tumor tissue showed a higher extent of necrosis in sections obtained from the B16F10 model treated with *A. vulgaris* extract in comparison to the control group (34.8 ± 16.5% and 20.4 ± 10.2%, respectively) (Figure 3A). On the other hand, although the volume of tumors was significantly reduced also in the B16F1 model, the degree of necrosis was unexpectedly higher in the control samples than in the treated group (37.3 ± 15.9% and 23 ± 7.7%, respectively), indicated that extract compounds, differently to B16F10 tumor model, did not reduce tumor size through induction of necrosis (Figure 3A). However, proliferating cell nuclear antigen (PCNA) expression, as the readout of cell mitosis rate, was significantly downregulated in these tumors upon the treatment of animals with *A. vulgaris* extract (Figure 3B). Most importantly, apart from the fact that tested plant extract did not promote apoptosis in B16F1 in vitro, in the tumor sections cell death areas, resembling apoptotic fields, were evident, additionally explaining shrunk tumor volume in lower grade solid melanoma model (Figure 3B). Namely, irregular nuclei shape, size, and chromatin condensation were noticed in all tissue samples of animals treated with *A. vulgaris* extract.

### 3.4. A. vulgaris Extract Enhances the Antitumor Immune Response in a Solid B16F1 Melanoma Model

Recently it was reported that apart from increased genetic instability in precancerous and cancerous lesions, tumors showed a tendency to decrease heterogeneity in high-grade forms by the establishment of well-functional, highly autonomous, and low immunogenic multicellular network [25,26]. Accordingly, in lower-grade stages, the immune system passes the route from pro-inflammatory to the establishment of an immunosuppressive protumorigenic environment. The presence of necrotic, necroptotic, and apoptotic cells in tumor tissue can lead to protumorigenic immune cell profiling through different pathways [27,28]. While apoptotic cells promoted the establishment of an anti-inflammatory milieu, necrotic cells released different molecules into the tumor microenvironment disabling the antitumor immune response. For this purpose, it was a challenge to investigate the potential of the tested extract to restore the host protective immunity against tumor and make a breakthrough in the immunosuppressive tumor microenvironment, apart from its direct effect on malignant cells.

**Figure 4:**
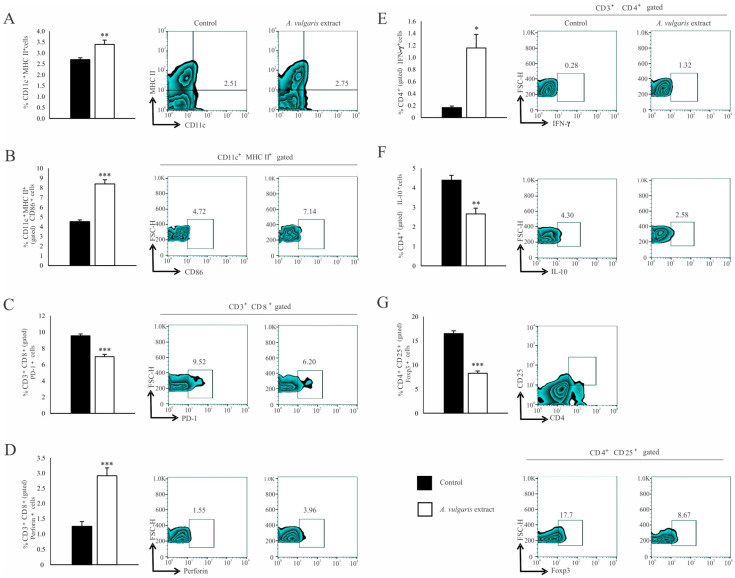
*A. vulgaris* extract alters the number and phenotype of immunocompetent cells in the spleen of tumor-bearing mice. The percentages of CD11c^+^MHCII^+^ (**A**), CD11c^+^MHCII^+^CD86^+^ (**B**), CD3^+^CD8^+^PD-1^+^ (**C**), CD3^+^CD8^+^perforin^+^ (**D**), CD3^+^CD4^+^IFN-γ^+^ (**E**), CD3^+^CD4^+^IL-10^+^ (**F**), CD4^+^CD25^+^Foxp3^+^ (**G**) cells in spleen were examined and presented in form of graphs and representative flow cytometry data (FACS) plots. In the experimental group, tumor-bearing mice were treated with *A. vulgaris* extract (i.p. 50 mg/kg in two cycles of 5 days with a break of two days), while the control group consisted of tumor-bearing mice treated with vehicle. Data are presented as means ± SEM of three individual experiments, each carried out with six mice per experimental group. Statistical significance was tested by Mann–Whitney rank-sum test or Student’s unpaired *t*-test and log-rank test where appropriate. * *p* < 0.05; ** *p* < 0.01; *** *p* < 0.001.

Significant increase in the percentage of CD11c^+^ dendritic cells (DCs) expressing MHC II (Figure 4A) and the costimulatory CD86 molecule (Figure 4B) in the spleen of the B16F1 melanoma bearing mice after treatment with *A. vulgaris* extract have been noticed. Although the analysis of functional T cell subpopulation did not reveal the difference in the percentage of CD3^+^CD8^+^ cells between the examined groups (Figure S10C), in the spleen of tumor-bearing mice treated with *A. vulgaris* extract, the percentage of PD-1 expressing CD8^+^ T cells was significantly decreased (Figure 4C). In addition, the percentage of perforin-producing CD3^+^CD8^+^ T cells was increased (Figure 4D). When it comes to Th cells, the percentages of splenic CD3^+^CD4^+^ T cells did not differ between groups (Figure S10B). However, a significantly higher percentage of interferon-gamma (IFN-γ) producing (Figure 4E) and a lower percentage of interleukin-10 (IL-10) producing CD4^+^ T cells (Figure 4F) derived from the spleen of tumor-bearing *A. vulgaris* extract treated mice were found. Importantly, the *A. vulgaris* extract remarkably decreased the percentage of CD3^+^CD25^+^Foxp3^+^ regulatory T cells (Tregs) in the spleens of tumor-bearing mice (Figure 4G).

**Figure 5:**
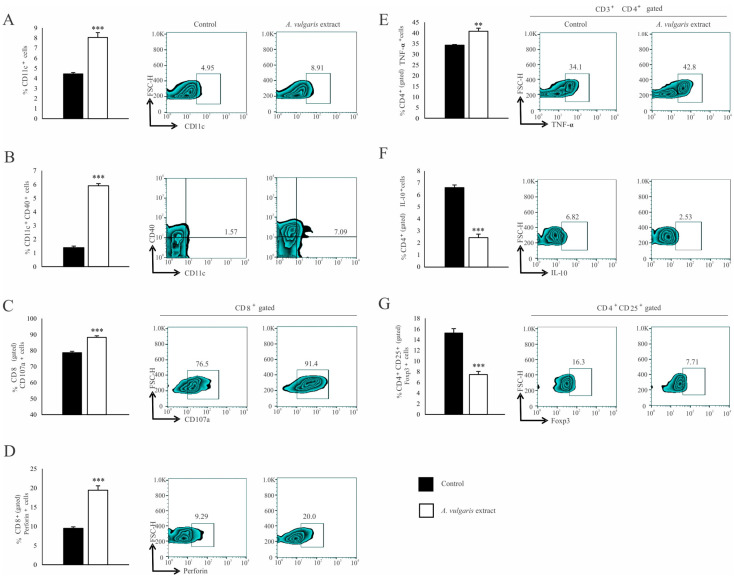
*A. vulgaris* extract enhances potent antitumor immune response in tumor microenvironment. The percentage of CD11c^+^ (**A**), CD11c^+^CD40^+^ (**B**), CD8^+^CD107a^+^ (**C**), CD8^+^perforin^+^ (**D**), CD4^+^TNF-α^+^ (**E**), CD4^+^IL-10^+^ (**F**), CD4^+^CD25^+^Foxp3^+^ (**G**), were analyzed and presented in form of graphs and representative flow cytometry data (FACS) plots. In the experimental group, tumor-bearing mice were treated with *A. vulgaris* extract (i.p. 50 mg/kg in two cycles of 5 days with a break of two days), while the control group consisted of tumor-bearing mice treated with a vehicle. Data are presented as means ± SEM of three individual experiments, each carried out with six mice per experimental group. Statistical significance was tested by Mann–Whitney rank-sum test or Student’s unpaired *t*-test and log-rank test where appropriate. ** *p* < 0.01; *** *p* < 0.001.

Similar results were observed in the analysis of tumor-infiltrating leukocytes (TILs). The *A. vulgaris* extract increased the percentage of total CD11c^+^ DCs (Figure 5A) as well as those expressing CD40 within that population (Figure 5B). The same treatment increased the percentage of CD3^+^CD8^+^ T cells (Figure S10F) in primary tumors. Furthermore, increments in the percentage of CD107a^+^ (Figure 5C), as well as perforin-producing CD8^+^ T cells (Figure 5D), in primary tumors derived from mice treated with *A. vulgaris* extract were detected. The percentage of CD3^+^CD4^+^ T cells in the tumor microenvironment was significantly increased after treatment with *A. vulgaris* extract (Figure S10E). The analyses of the functional phenotype of CD3^+^CD4^+^ T cells revealed an increment of the percentage of tumor necrosis factor-α (TNF-α) producing (Figure 5E) and a decrement of IL-10 producing CD4^+^ cells (Figure 5F), after treatment with *A. vulgaris* extract. Finally, the same treatment abrogated CD4^+^CD25^+^Foxp3^+^ Treg percentage in the tumor (Figure 5G), opening the space for reestablishment of active antitumor immune response.

### 3.5. A. vulgaris Extract Realized a Strong Antitumor Effect in Both Melanoma Models without Remarkable Systemic Toxicity

**Figure 6:**
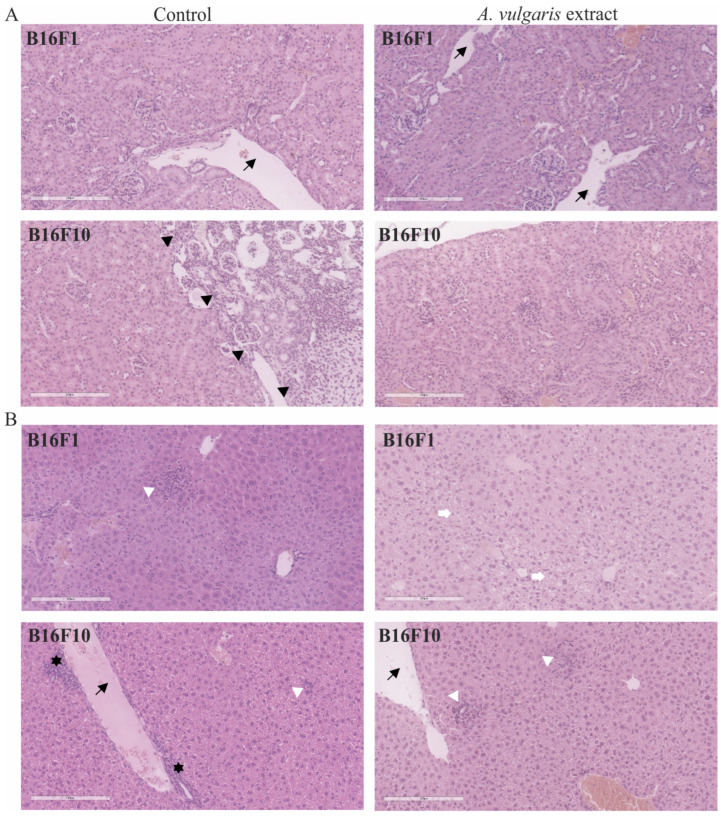
Representative micrographs of the most significant histopathological alterations of kidney (**A**) and liver (**B**) were noted in B16F1 and B16F10 models. Black arrows point to dilated venules. Scale bar 200 µm. Black arrowheads delineate immature kidney tissue on the right from mature on the left. White arrowheads show foci of inflammatory infiltrates in liver tissue. White arrows mark vacuolated hepatocytes. Black stars reveal extramedullary hematopoiesis. All pictures are H/E stained and 200× magnified.

Apart from the evident antitumor potential of *A. vulgaris* in both forms of solid melanoma, realized through the interplay between direct effect against tumor cells and tumor microenvironment, its influence on healthy tissue, especially those involved in metabolism and clearance, can be of leading importance for the outcome of the treatment. For that purpose, in addition to the tumor tissue, histopathological changes in the kidney (Figure 6A) and liver (Figure 6B) were microscopically estimated. Discrete and no significant changes were noticed in samples collected from the animals exposed to the treatment. Only a few scant mononuclear interstitial infiltrates and dilatation of the cortical venules in kidney tissue samples together with the rare tubules with protein casts were identified. The analysis of dilation of portal venules, vacuolization of periportal hepatocytes, scant foci of extramedullary hematopoiesis, and areas of mononuclear cell infiltration revealed no significant differences between groups. These results presented the first line of evidence supporting the hypothesis that the tested extract did not promote significant toxicity and are in concordance with the data obtained from urine parameter assessment in animals exposed to the treatment (Table S1). Taken together, *A. vulgaris* extract realized a strong antitumor effect in both melanoma models without evident systemic toxicity.

## 4. Discussion

Our previous study has shown that *A. vulgaris* ethanolic extract is rich in phenolic compounds and exhibits strong anticancer potential [17]. While numerous studies have delved into the biological activity of isolated molecules, it is apparent that their interactions exert diverse effects. These interactions can not only impact tumor cells but also influence other components of the tumor microenvironment, including immune and stromal cells, blood vessels, etc. The well-expressed synergy among bioactive compounds often results in crude extracts exhibiting higher biological effects than their individual components. However, the serious obstacle of crude extract application is the inconsistence in its composition due to geographic and sessional variation resulting in low reproducibility of obtained data. This makes difficulties in extract standardization and manufacturing on a large scale representing insurmountable barriers for eventual usage in clinical practice [29,30]. On the other hand, the correlation between content and biological activity can give valuable insight into the interaction of compounds with different anticancer mechanisms and how they contribute to the observed effects. The example of agents that were included in clinical practice, so-called vinca alkaloids, vinblastine (VLB), and vincristine (VCL), were isolated from the vinca plant (*Catharanthus roseus* (L.) G. Don. (Apocynaceae)). It was observed that the extract of this plant reduces the number of white blood cells as well as the number of hematopoietic cells in the bone marrow of rats, and later the activity against lymphocytic leukemia in mice was also determined. These studies led to the isolation of VLB and VCL as active components, and their discovery can be indirectly ascribed to the original research on the activity of this plant extract [31]. Given the rich ethnomedicinal history of the *A. vulgaris* extract, it is intriguing to explore the impact of a mixture of bioactive compounds when they are applied in a well-defined ratio balanced by nature. Our objective was to validate these ethnopharmacological data which can serve as an initial point for further comprehensive analyses. Collected results can serve as a basis for designing combined protocols where the use of different isolated components can help in overcoming the therapy resistance.

Unlike previous qualification of tumors as diseases of random behavior, today we face the still unexplainable strategy of intra- and extra-tumoral communications conducted by perfect multicellular orchestration, the complexity of which is only “touched” in the recent years of research [32]. From this platform, the limited response of high-grade tumors to therapy is not only a consequence of the establishment of apoptotic-resistant phenotype but also an intercellular communication in the microenvironment leading to tumor progression. The net effect of these interactions is reflected in the initial or acquired therapy resistance, resulting in tumor progression and repopulation upon the treatment [33,34].

However, there are serious deficiencies in conclusions that arise from experiments performed in cell culture related to oversimplified system and lack of the complexity of neoplastic diseases when they are judged in the context of the tumor microenvironment and influence on disease-affected tissues [35]. The problem of deficiencies is not completely solved by in vivo models whether it is a syngeneic, xenograft, or patient-derived xenograft since each of them possesses its own imperfections [36]. To explore the antimelanoma potential of *A. vulgaris* extract in the context mentioned above, in this study its effects were evaluated in vitro and in vivo, using syngeneic models of melanoma of less and higher invasive forms. This allowed us to get insight into major differences in response to applied treatment of the melanoma primary tumor depending on the grade and, accordingly, aggressiveness in the context of preserved immune system that exist only in syngeneic models. Sensitivity observed in vitro when F1 and F10 cells were exposed to *A. vulgaris* extract was highly reproducible in vivo, measured by a noteworthy reduction of tumor volume in both primary tumors, but with one remarkable difference. While in vitro response of the F1 clone to *A. vulgaris* extract was significantly higher in comparison to F10, as expected regarding the stemness of the last, in vivo treatment resulted in obvious tumor shrinkage in both models but with a more prominent effect in high-grade F10 form. This observation underlines the specificity of Lady’s mantle extract to exert an even more profound antitumor effect in the high-grade state of the disease, but only when it is assessed in vivo, indicating the importance of all constituents of tumor tissue and its features. Histopathological assessment of the tumors in both models revealed that *A. vulgaris* treatment led to enhanced necrosis in advanced tumors, while this effect was not present in the tumor model induced by subcutaneous inoculation of F1 generation of B16 cells. On the other hand, in tumor tissue large apoptotic surfaces and significantly reduced mitotic index in the surrounding area were found. Flow cytofluorimetric evaluation of B16F1 and B16F10 cells in vitro exposed to *A. vulgaris* extract revealed significant deviations from the in vivo data. Apart from a well-synchronized decrease in proliferation in vitro and in vivo, the complete absence of apoptosis was obvious in B16F1 cultures in vitro, while in tissue samples large apoptotic-like areas triggered by the treatment were evident. Significant necrosis promoted by *A. vulgaris* extract in samples obtained from the B16F10 model was in discrepancy with dominant apoptotic cell death determined in vitro in cell culture exposed to extract. Unlike the cell culture, in the tissue apoptotic cells are eliminated by phagocytosis in an immunosuppressive manner. Furthermore, massive apoptosis in tumor tissue, which basically presented a major goal of applied radio- or chemotherapy, if not balanced with other signals that can limit anti-inflammatory and mitogen stimuli delivered from direct and indirect intercellular communication in apoptotic-rich areas, promotes tumor progression, immune escape, and repopulation in response to the chemo- or radiotherapy [37]. Partly because of this, the overall impression is that herbal extracts, oppositely to well-defined effects of nonselective chemotherapeutics, affect the tumor microenvironment at multiple levels regarding the content of synergistically active compounds. This opens the possibility to influence tumor cell growth, but in parallel prepares tumor tissue for a host-protective response against malignant phenotypes. 

Recently it was reported that genetic instability and tumor heterogeneity increase in precancerous and cancerous lesions but decrease with tumor grade, indicating that the key period for immune response as well as immune escape and establishment of immunosuppressive environment is defined at lower grade phase [25,26]. This further points to the fact that in this stage of the disease, the activity of the immune response and, consequently, the further course of the disease can be influenced by the treatment with herbal extract as *A. vulgaris* is. Concordantly, the assessment of the tumor microenvironment and spleen cellular makeup showed that *A. vulgaris* extract led to a turnaround and re-establishment of immune cells’ antitumor activities. Mature DCs are the most important cells in initiating and regulating acquired immune responses to growing tumors and, in parallel, one of the common targets of tumor cells’ immunosuppressive activities [38,39]. Decreased immunogenicity occurs because of the lack of T cell stimulation [40], loss of MHC class I expression [41,42], inadequate antigen presentation of CD4^+^ to helper cells [43] as well as production of immunosuppressive factors [44,45]. DCs exposed to *A. vulgaris* extract, begin to express significantly more MHC II, costimulatory CD86 molecules systemically and express more CD40 ligand locally, thus becoming competent to correctly display tumor antigens and activate the T cell response.

Although it has been shown that CD4^+^ T cells specific for tumor antigens can mediate the elimination of tumor cells even in the absence of endogenous expression of MHC class II on tumor cells, the presence of the mature potent DCs is extremely important for their activation [46]. After activation, CD4^+^ T cells differentiate into Th1-type cells producing high levels of Th1 cytokines, which has been shown to be a major mediator in tumor control [47,48]. Results presented in this study showed that activated CD4^+^ T cells differentiate into Th1 direction and produce more IFN-γ in the spleen due to *A. vulgaris* extract treatment. IFN-γ can directly interact with tumor cells [49], or indirectly promote the differentiation of CTL effectors [50]. Concomitantly, IFN-γ can facilitate CTLs to enter the effector site. Increased accumulation of TNF-α producing CD4^+^ Th cells in the tumor microenvironment was also observed in mice after treatment. It is possible that TNF-α increases the susceptibility of target cells to T cell-dependent cytolysis and thereby contributes to and accelerates target cell death [51]. In line with our findings, the production of TNF-α is essential for the elimination of melanoma by Th1 cells [52]. Besides the stimulatory effect of *A. vulgaris* extract on DCs maturation and Th1 cell differentiation, the decrement in the accumulation of immunosuppressive IL-10-producing Th cells and Tregs in the spleen and primary tumor were detected. This effect greatly contributes to an immune response and more successful suppression of tumor growth. It is known that Treg cells play a crucial role in immune suppression by tumor-specific T cell response inhibition, while their infiltration into the tumor is strongly associated with tumorigenesis [53,54]. Importantly, cytotoxic CD8^+^ T cells versus CD4^+^Foxp3^+^ regulatory T cells ratio may have predictive value for melanoma outcome [55].

An important component of immunosuppression and immune cell tolerance to tumor cells is closely connected with the programmed death ligand-1/programmed death-1 (PD-L1/PD-1) signaling pathway [45]. Although cytotoxic T lymphocytes (CTLs) can recognize tumor-associated antigens expressed on tumor cells, they are generally not capable of inducing a productive immune response due to PD-1 mediated negative signaling through the immunoreceptor tyrosine switch (ITSM) [56,57]. DCs as potent antigen-presenting cells activate CD8^+^ T cells. This study showed that *A. vulgaris* extract significantly increases the accumulation of cytotoxic lymphocytes in the tumor microenvironment with a reduced percentage of those expressing the inhibitory molecule PD-1 on their surface. CD107a (also known as LAMP-1) is a surrogate marker of cytotoxic activity expressed by cytotoxic T cells, natural killer (NK), and γδ T cells, which appears on the cell surface in a short time interval after degranulation. CD107a expression is closely related to IFN-γ and TNF-α expression and T cell cytotoxicity [58]. In concordance with the diminished PD-1 expression, Increased expression of markers for cytotoxic activity- CD107a and perforin in CD8^+^ T cells derived from the spleen and primary tumor triggered by the extract treatment strongly support the hypothesis about the re-establishment of successful antitumor immune response. 

It seems that bioactive compounds from *A. vulgaris* facilitate DCs maturation and subsequent Th1 and CTL differentiation. In brief, extract treatment supports antitumor immunity in at least two ways. TNF-α producing Th cells and tumoricidal CD8^+^ T cells migrate into the tumor site, contact tumor cells, and release perforin that mediates killing cancer cells. In parallel, inhibited accumulation of immunosuppressive cells in the tumor microenvironment additionally facilitates the development of potent antitumor immune response. All the above leads us to the conclusion that the *A. vulgaris* extract significantly modulates the immune response to the tumor in favor of Th1 lymphocytes and cytotoxic mediators, whose activity can successfully suppress the growth and spreading of the tumor. 

There are still debates about the possible cytotoxicity of the tannin compounds [59], where ellagitannins were identified as significant constituents in the Alchemillae herba [60]. The controversial data mainly refer to the liver-targeted toxicity effects of the tannin-rich extracts [61]. Nevertheless, no cytotoxic effects on breast, liver, and kidney cells caused by Alchemilla extract rich in tannin fraction were observed in our recent ex vivo animal model experiments [62]. Together with the fact that treatment is well tolerated by healthy tissues, supporting the host’s overall potential to fight against the disease, the anticancer potential of this plant is worthy of further preclinical investigation.

## 5. Conclusions

Apart from the impressive improvement that targeted, and immunotherapy brought to the advanced melanoma treatment, the success is still limited by initial or acquired resistance mechanisms, thus opening the space for complementary or alternative approaches in the treatment of oncology patients. The results of this study revealed two-dimensional benefits of the application of *A. vulgaris* extract in melanoma of different grades—persistent and even deeper ability to reduce high-grade tumors and, in parallel, strong re-establishment of antitumor immunity through stimulation of the innate and adaptive antitumor immune response and restriction of the immunosuppressive environment of low-grade tumors. Overall, this is the first report on the anticancer activity of the extract of *A. vulgaris* on a melanoma model in vivo, which provides evidence of its multilevel potential and efficacy in advanced melanoma, thus strongly supporting the importance of further research in this area.

## Figures and Tables

**Table 1:** IC_50_ values of *A. vulgaris* extract on melanoma cells.

Cell Lines	Assay	IC50 [µg/mL]
B16F1	MTT	34.3 ± 4.2 ^1^
	SRB	43.2 ± 0.1
B16F10	MTT	72.2 ± 2.7
	SRB	80.9 ± 1.0
518A2	MTT	62.75 ± 6.5
	SRB	88.55 ± 6.29
Fem-X	MTT	78.4 ± 5.20
	SRB	90.3 ± 8.49

^1^ mean ± SD.

## Data Availability

Data supporting obtained results can be obtained from the authors upon request.

## Supplementary Materials

The following supporting information can be downloaded at: https://www.mdpi.com/article/10.3390/diseases12060125/s1, Figure S1. *A. vulgaris* effect on melanoma cell viability in vitro. Figure S2. *A. vulgaris* extract effect on murine embryonic fibroblasts (NIH/3T3), and human immortalized keratinocytes (HaCaT). Figure S3. Representative flow cytometry data in B16F1 (A) and B16F10 (B) cell lines. Figure S4. *A. vulgaris* extract influence on cell cycle distribution of both- B16F1 (A) and B16F10 (B) cell lines. Figure S5. *A. vulgaris* extract effect on the nuclei morphology of PI stained B16F1 (A) and B16F10 (B) cells. Figure S6. *A. vulgaris* extract influence on total caspase activation in both- B16F1 (A) and B16F10 (B) cell lines. Figure S7. Flow cytometry analysis of *A. vulgaris* extract-treated B16F1 cells after LysoTracker Red staining. Figure S8. Cell viability assessment in concomitant treatment with *A. vulgaris* extract and GSH or NAC. Figure S9. ROS/RNS production in concomitant treatment with *A. vulgaris* extract and GSH or NAC. Figure S10. *A. vulgaris* effect on immune response in spleen and tumor tissue. Figure S11. Tumor growth curve B16F1. Table S1: Mouse urine parameters.
