# Supplementary material for: Antimelanoma Effects of Alchemilla vulgaris: A Comprehensive In Vitro and In Vivo Study
Source: Diseases. 2024 Jun 8;12(6):125. doi: 10.3390/diseases12060125 (PMC11202689; doi:10.3390/diseases12060125)
Supplement: Supplementary file 1 [file diseases-12-00125-s001.zip › diseases-3015644-supplementary.pdf]

SUPPORTING INFORMATION:

Article

Antimelanoma Effects of *Alchemilla vulgaris*: A Comprehensive *In Vitro* and *In Vivo* Study

Sanja Jelača [1], Ivan Jovanovic [2], Dijana Bovan [1], Sladjana Pavlovic [2], Nevena Gajovic [2], Duško Dunđerović [3], Zora Dajić-Stevanović [4], Aleksandar Acović [5], Sanja Mijatović [1,*], Danijela Maksimović-Ivanić [1,*]

[1] Department of Immunology, Institute for Biological Research "Siniša Stanković" – National Institute of the Republic of Serbia, University of Belgrade, Bulevar Despota Stefana 142, 11108 Belgrade, Serbia; sanja.jelaca@ibiss.bg.ac.rs (S.J.); dijana.draca@ibiss.bg.ac.rs (D.B.)
[2] Center for Molecular Medicine and Stem Cell Research, Faculty of Medical Sciences, University of Kragujevac, Svetozara Markovića 69, 34000 Kragujevac, Serbia; ivanjovanovic77@gmail.com (I.J.); sladjadile@gmail.com (S.P.); gajovicnevena@yahoo.com (N.G.)
[3] Institute of Pathology, School of Medicine, University of Belgrade, Dr Subotića 8, 11000 Belgrade, Serbia; dusko.dundjerovic@med.bg.ac.rs
[4] Faculty of Agriculture, University of Belgrade, Nemanjina 6, 11080 Belgrade, Serbia; dajic@agrif.bg.ac.rs
[5] Department of Dentistry, Faculty of Medical Sciences, University of Kragujevac, Svetozara Markovića 69, 34000 Kragujevac, Serbia. dr.acovic115@gmail.com
* Correspondence: (D.M-I.) nelamax@ibiss.bg.ac.rs; Tel.: +381-11-2078452, Bulevar Despota Stefana 142, 11108 Belgrade, Serbia; (S.M.) sanjamama@ibiss.bg.ac.rs; Tel.: +381-11-2078452, Bulevar Despota Stefana 142, 11108 Belgrade, Serbia

List of content:

Table S1: Mouse urine parameters.

Figure S1. *A. vulgaris* effect on melanoma cell viability *in vitro.*

Figure S2. *A. vulgaris* extract effect on murine embryonic fibroblasts (NIH/3T3), and human immortalized keratinocytes (HaCaT).

Figure S3. Representative flow cytometry data in B16F1 (A) and B16F10 (B) cell lines.

Figure S4. *A. vulgaris* extract influence on cell cycle distribution of both- B16F1 (A) and B16F10 (B) cell lines.

Figure S5. *A. vulgaris* extract effect on the nuclei morphology of PI stained B16F1 (A) and B16F10 (B) cells.

Figure S6. *A. vulgaris* extract influence on total caspase activation in both- B16F1 (A) and B16F10 (B) cell lines.

Figure S7. Flow cytometry analysis of *A. vulgaris* extract-treated B16F1 cells after LysoTracker Red staining.

Figure S8. Cell viability assessment in concomitant treatment with *A. vulgaris* extract and GSH or NAC.

Figure S9. ROS/RNS production in concomitant treatment with *A. vulgaris* extract and GSH or NAC.

Figure S10. *A. vulgaris* effect on immune response in spleen and tumor tissue.

Figure S11. Tumor growth curve B16F1.

Table S1. Mouse urine parameters.

[1] mean ± SD.

	B16F1		B16F10	
	**Control**	***A. vulgaris* extract**	**Control**	***A. vulgaris* extract**
Spec. gravity	1028.3 ± 2.6 [1]	1030 ± 0	1029.6 ± 1.4	1028.3 ± 2.6
pH	5.7 ± 0.5	6 ± 0	6.1 ± 0.3	6.1 ± 0.2
Glucose (mg/100mL)	negative	negative	negative	negative
Nitrite	negative	negative	negative	negative
Protein (mg/100mL)	22.5 ± 8.2	26.3 ± 7.5	10.3 ± 12.5	10 ± 0
Ketones (mg/100mL)	10 ± 0	11.7 ± 5.8	8.8 ± 2.3	7.5 ± 2.7
Urobilinogen (mg/100mL)	0.4 ± 0.4	0.4 ± 0.4	2.3 ± 1.5	2 ± 1.5
Bilirubin (mg/100mL)	0.4 ± 0.1	0.5 ± 0	0.6 ± 0.2	0.58 ± 0.2
Blood (RBC/μL)	negative	negative	negative	negative
Leukocytes (WBC/μL)	negative	negative	negative	negative

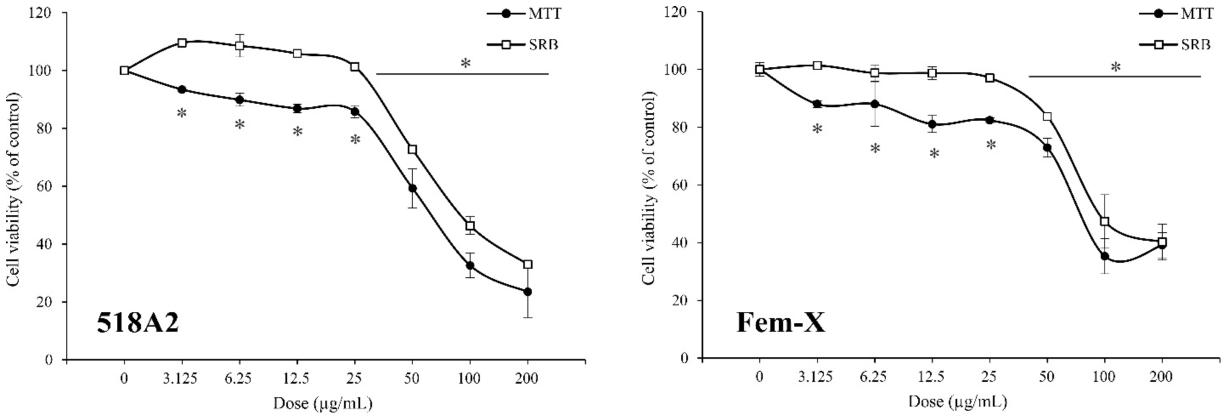

Figure S1. *A. vulgaris* effect on melanoma cell viability *in vitro.* 518A2 and Fem-X cells were treated with a wide range of concentrations of *A. vulgaris* extract and viability assays (MTT and SRB) were performed after 72 h. All data are presented as mean ± SD from one representative out of three independent experiments and statistically significant were considered p values less than 0.05, comparing to controls.

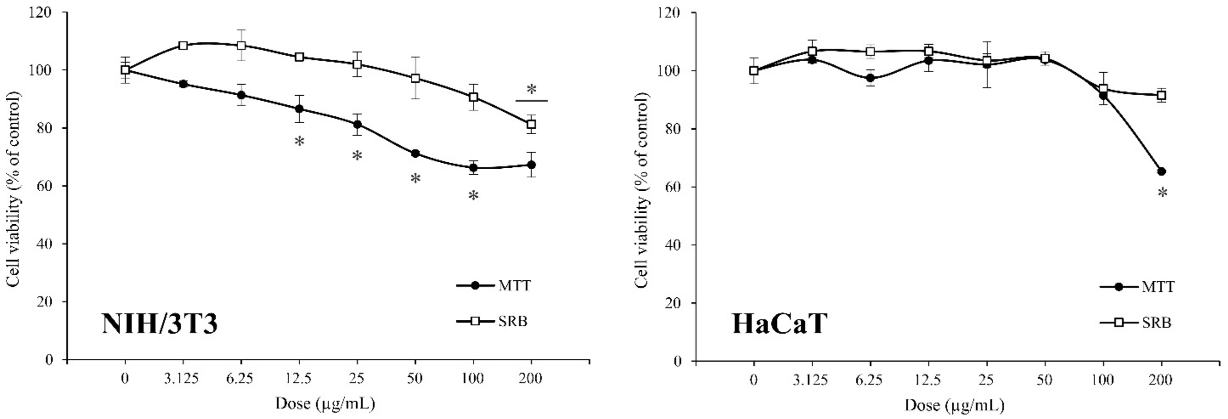

Figure S2. *A. vulgaris* extract effect on murine embryonic fibroblasts (NIH/3T3), and human immortalized keratinocytes (HaCaT). Cells were treated with a wide range of concentrations of *A. vulgaris* extract. MTT and SRB assays were performed after 72 h. Data are presented as mean ± SD from one representative out of three independent experiments and statistically significant were considered *p* values less than 0.05, comparing to controls.

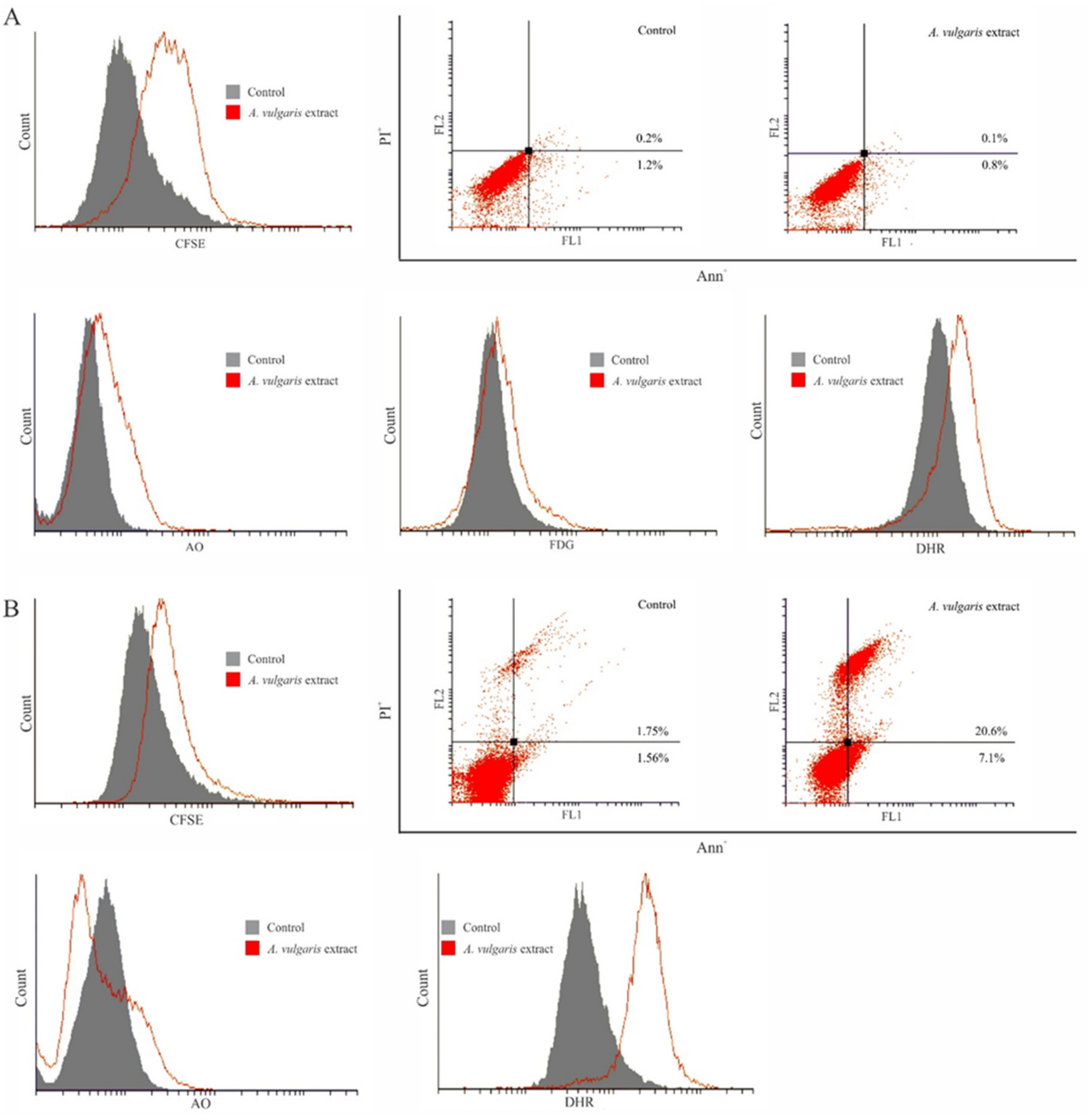

Figure S3. Representative flow cytometry data in B16F1 (A) and B16F10 (B) cell lines. Cells were treated with an IC_{50} concentration of *A. vulgaris* extract for 72 h. Cellular proliferation (CFSE), apoptosis (Ann/PI), autophagy (AO), senescence (FDG), and production of ROS/RNS species (DHR) were detected by corresponding staining followed by flow cytometry analysis.

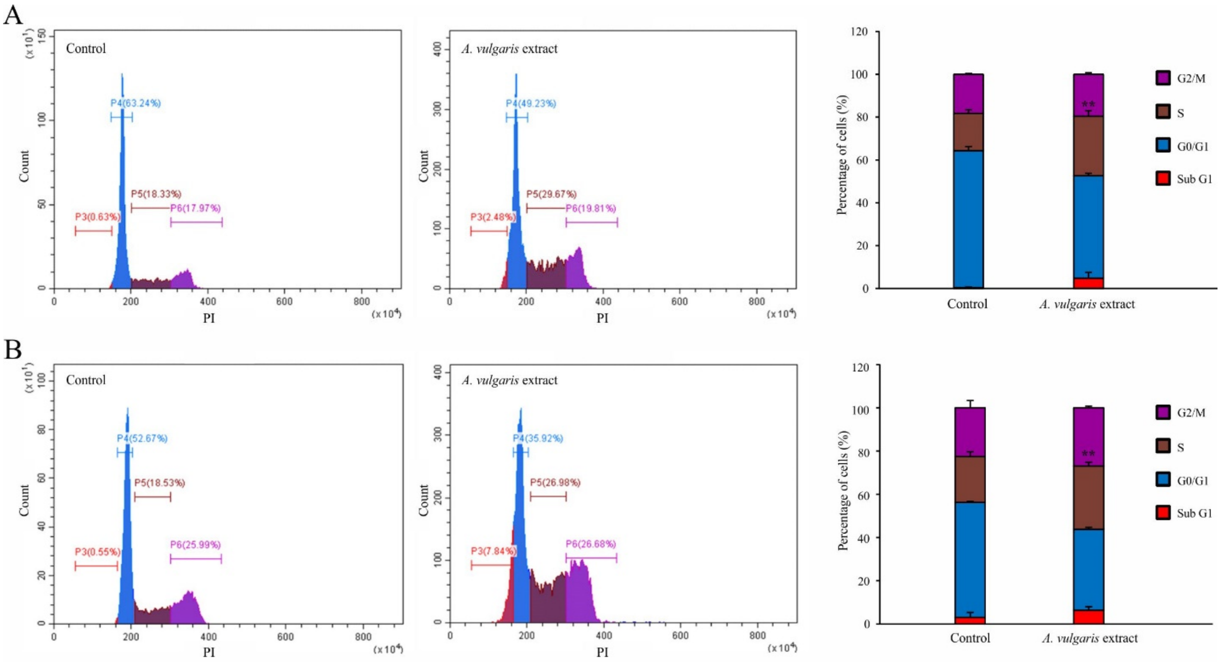

Figure S4. *A. vulgaris* extract influence on cell cycle distribution of both- B16F1 (A) and B16F10 (B) cell lines. Cells were treated with an IC_{50} dose of *A. vulgaris* extract for 72 h. Distribution of B16F1 and B16F10 cells within cell cycle phases was evaluated using PI staining followed by flow cytometry analysis. Representative flow cytometry data from one and mean ± SD from three independent experiments are presented. ** $p < 0.01$ comparing to control.

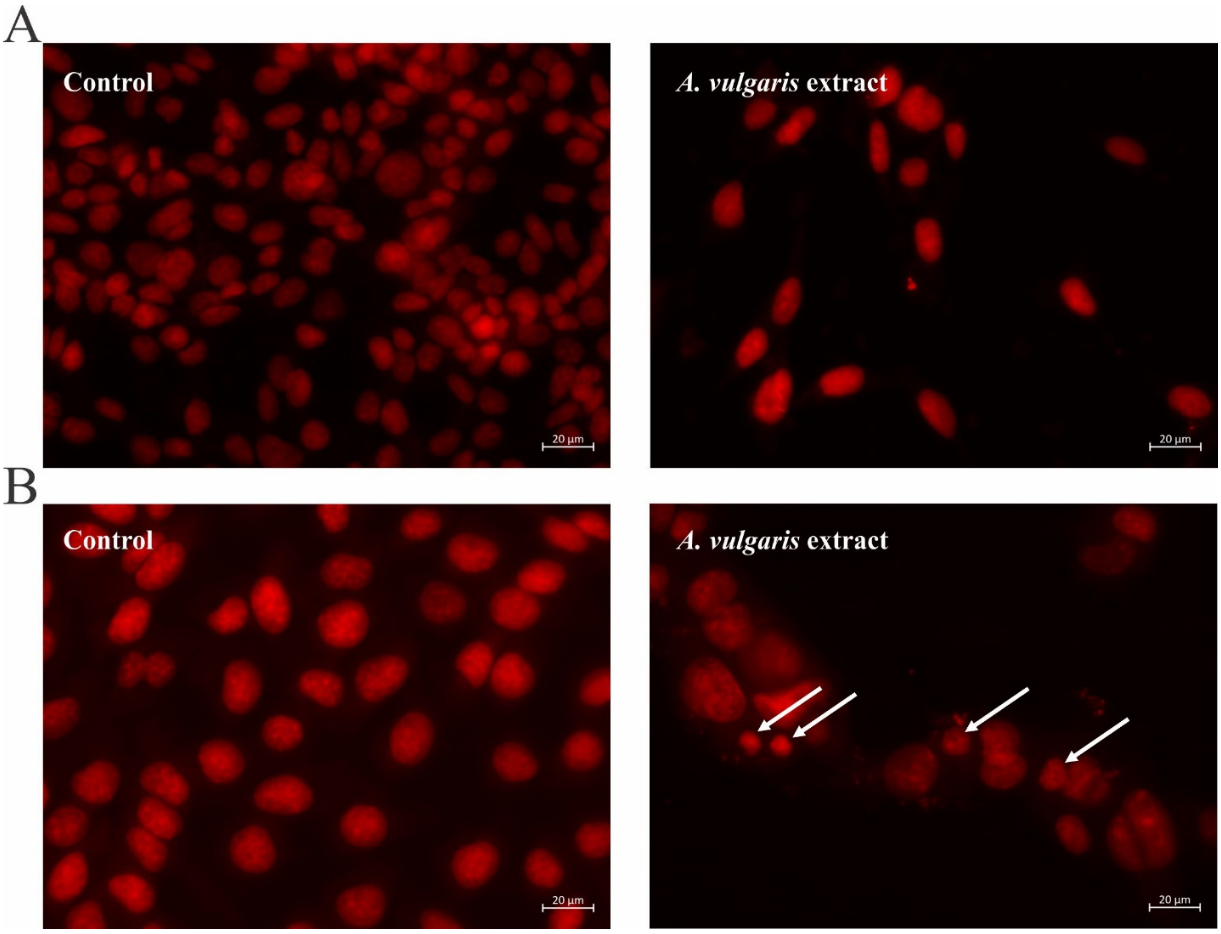

Figure S5. *A. vulgaris* extract effect on the nuclei morphology of PI stained B16F1 (A) and B16F10 (B) cells. Both cell lines were treated with an IC_{50} dose of *A. vulgaris* extract for 72 h. Cells were stained with PI staining and analyzed using fluorescence microscopy. White arrows mark apoptotic nucleus.

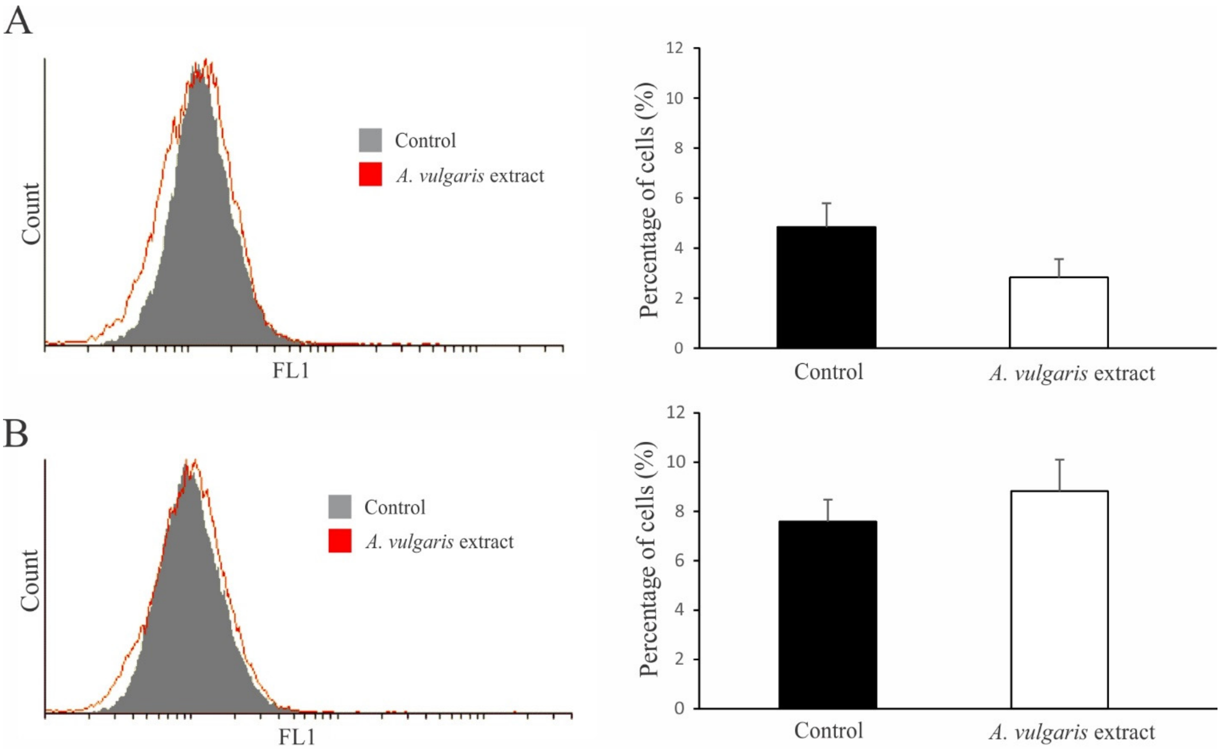

Figure S6. *A. vulgaris* extract influence on total caspase activation in both- B16F1 (A) and B16F10 (B) cell lines. Cells were treated with an IC_{50} dose of *A. vulgaris* extract for 72 h. Caspase activation was detected by apostat staining followed by flow cytometry analysis. Representative flow cytometry data from one and mean ± SD from three independent experiments are presented.

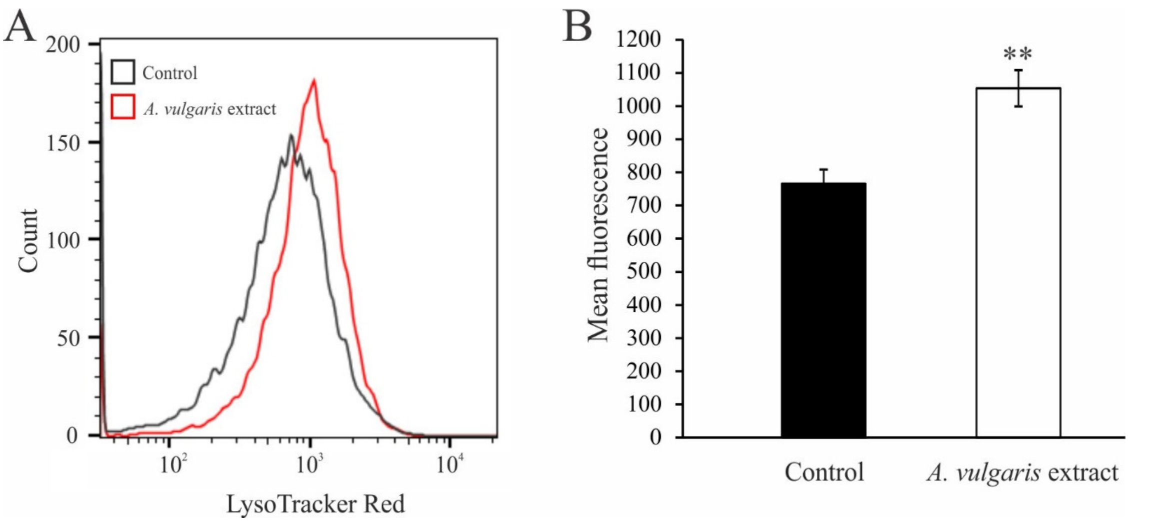

Figure S7. Flow cytometry analysis of *A. vulgaris* extract-treated B16F1 cells after LysoTracker Red staining. Cells were treated with an IC_{50} dose of *A. vulgaris* extract for 72 h and LysoTracker Red staining was performed, followed by flow cytometry analysis. Representative flow cytometry data from one (A) and mean ± SD from three independent experiments (B) are presented. ** $p < 0.01$ comparing to control.

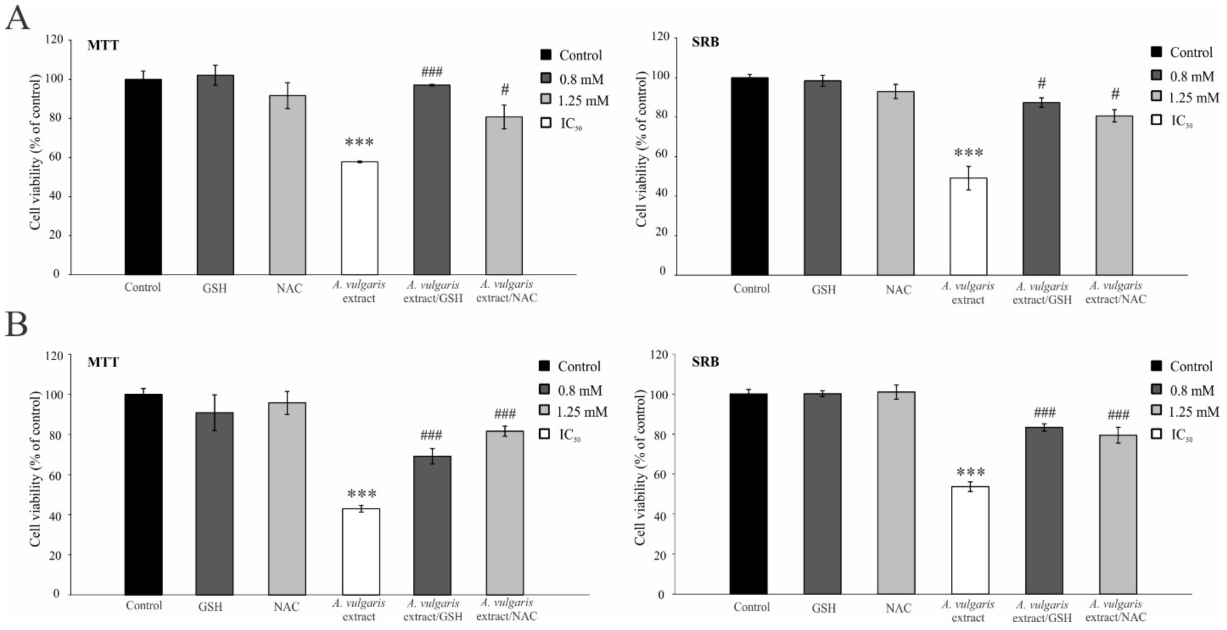

Figure S8. Cell viability assessment in concomitant treatment with *A. vulgaris* extract and GSH or NAC. B16F1 (A) and B16F10 (B) cell viability after combined treatment with *A. vulgaris* extract and antioxidants GSH (0.8 mM) or NAC (1.25 mM) was assessed by MTT and SRB assay. Data are presented as mean ± SD from one representative out of three independent experiments. *** $p < 0.001$ comparing to control and # $p < 0.05$; ## $p < 0.01$; ### $p < 0.001$ comparing to *A. vulgaris* extract treatment.

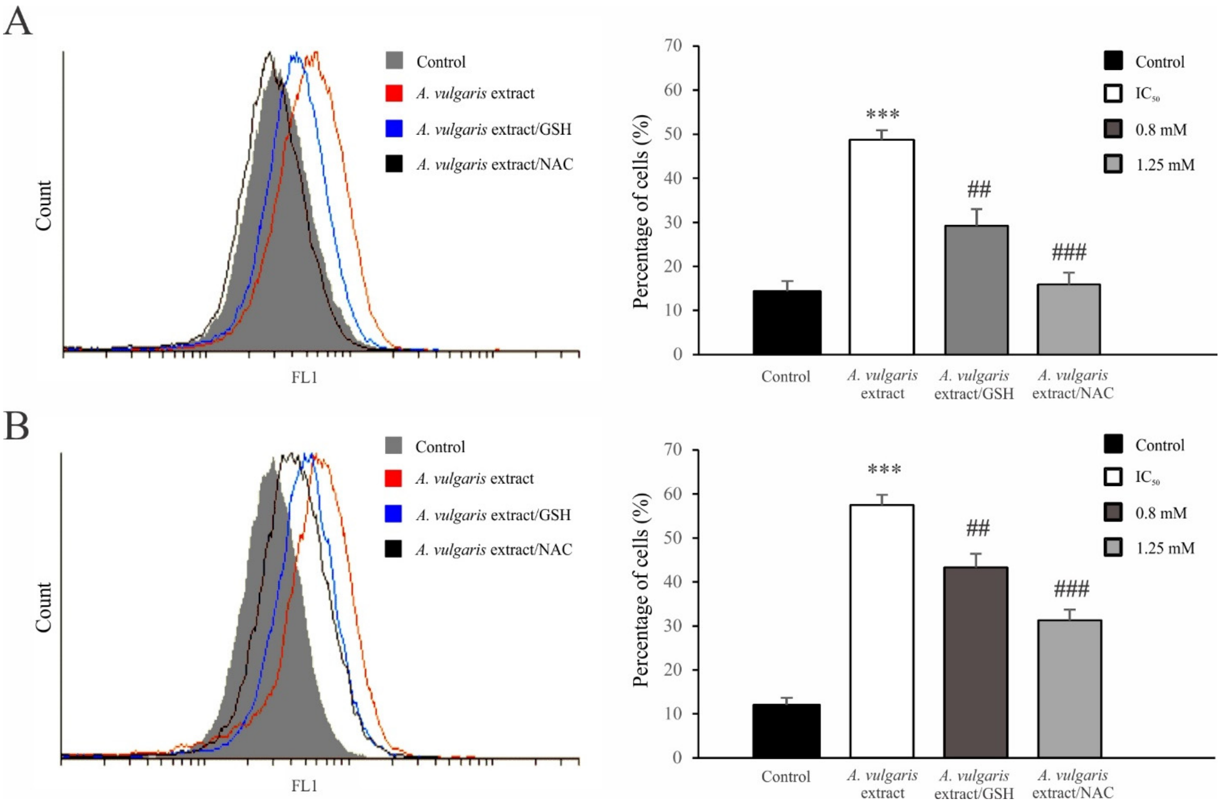

Figure S9. ROS/RNS production in concomitant treatment with *A. vulgaris* extract and GSH or NAC. B16F1 (A) and B16F10 (B) cells were pretreated with DHR staining and treated with an IC_{50} concentration of *A. vulgaris* extract alone and in concomitant treatment with antioxidants GSH (0.8 mM) or NAC (1.25 mM) for 72 h. Production of ROS/RNS species was detected by flow cytometry analysis. Representative flow cytometry data from one and mean ± SD from three independent experiments are presented. *** $p < 0.001$ comparing to control and ## $p < 0.01$; ### $p < 0.001$ comparing to *A. vulgaris* extract treatment.

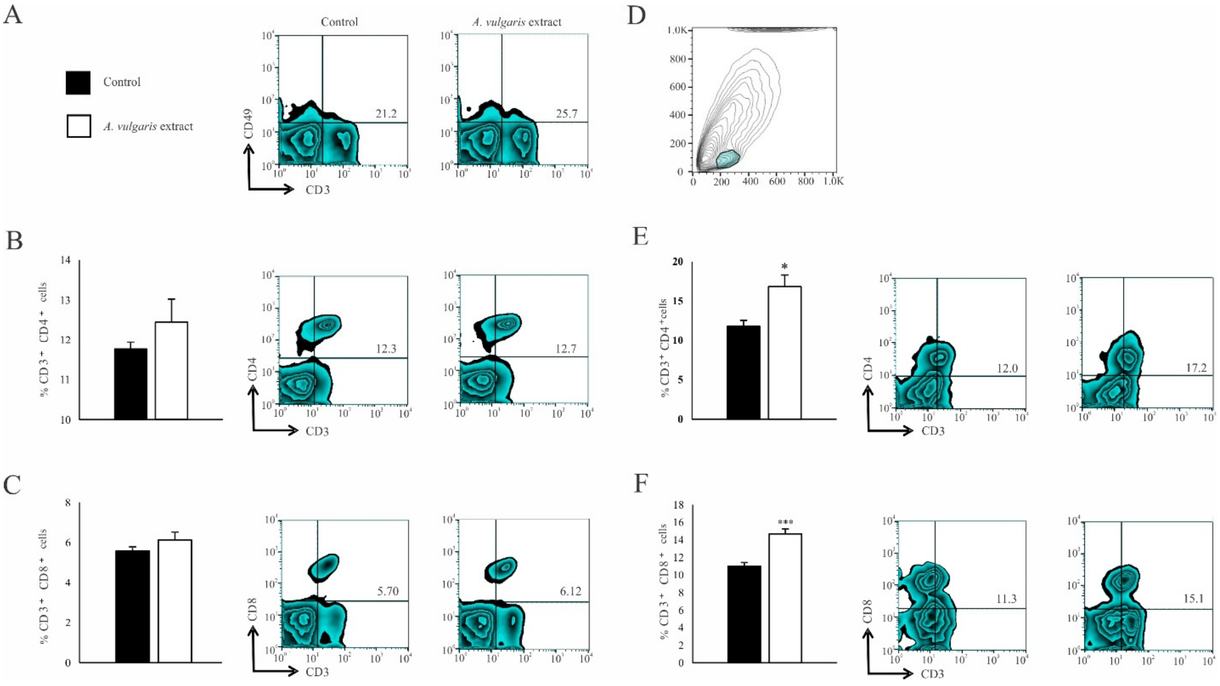

Figure S10. *A. vulgaris* effect on immune response in spleen and tumor tissue. Graph A shows the percentage of CD49b⁺CD3⁺ T cells in the spleen. Graph B shows the percentage of CD3⁺CD4⁺ T cells in the spleen. Graph C shows the percentage of CD3⁺CD8⁺ T cells in the spleen. Graphs D illustrate gating strategy for mononuclear cells in tumor microenvironment. Graph E shows the percentage of CD3⁺CD4⁺ T cells in primary tumors. Graph F shows the percentage of CD3⁺CD8⁺ T cells in primary tumors. Statistical significance was tested by Mann–Whitney Rank Sum test (B) or Student's unpaired *t*-test (A, B, C, E, and F).

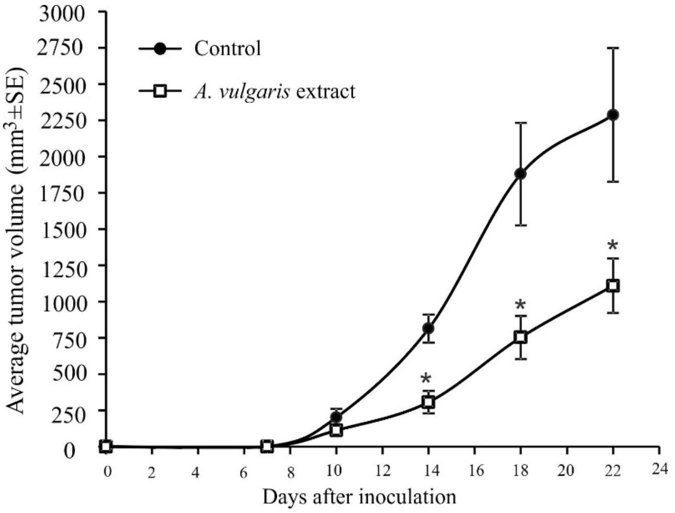

Figure S11. Tumor growth curve B16F1. Tumors were induced by subcutaneous implantation of B16F1 cells into right dorsal lumbosacral region of C57BL/6 mice. Treatment with *A. vulgaris* extract started when tumors became palpable (day 7) and tumor volume was measured in the indicated time points. Average tumor volume (mm^3) ± SE were presented and p values of less than 0.05 were considered statistically significant (*).
